# Broccoli Sprout Extract Restores Renal Lipid Metabolism in High‐Fat Diet‐Fed Mice Involving Modulation of AMPK/Nrf2 Signaling and the NLRP3 Inflammasome

**DOI:** 10.1002/fsn3.72348

**Published:** 2026-09-21

**Authors:** Chunting Wu, Bangzhao Zeng, Xuexun Li, Xin Zhao, Junyu Ma, Mengxue Zhang, Shuangshuang Zhao, Chunmei Zhang, Qin Gao, Fuli Ya

**Affiliations:** ^1^ Department of Nutrition, School of Public Health Dali University Dali China; ^2^ Department of Laboratory Teaching Center, School of Public Health Dali University Dali China; ^3^ School of Public Health Jining Medical University Jining China

**Keywords:** AMPK, broccoli sprout extract, high‐fat diet, lipid metabolism, NLRP3 inflammasome, Nrf2, renal injury

## Abstract

Broccoli sprout extract (BSE) has shown promise in metabolic disorders, but its effect on renal lipid metabolism and injury in high‐fat diet (HFD)‐fed mice remains unclear. This study demonstrated that 12‐week BSE supplementation (600 mg/kg diet) significantly ameliorated HFD‐induced renal dysfunction, reducing blood urea nitrogen (BUN) from 6.29 to 3.02 mmol/L (by 52.0%), uric acid (UA) from 56.38 to 17.25 μmol/L (by 69.4%), and serum creatinine (SCr) from 19.21 to 11.54 μmol/L (by 39.9%), while decreasing renal triglycerides (TG) from 3.86 to 2.43 mmol/g (by 37.1%) and total cholesterol (TC) from 19.47 to 14.43 mmol/g (by 25.9%). BSE also restored oxidative balance, increasing superoxide dismutase (SOD) activity from 4.37 to 6.54 U/mg protein (by 49.7%) and total antioxidant capacity (T‐AOC) from 1.78 to 2.97 μmol/mg protein (by 66.9%), with a reduction in malonaldehyde (MDA) from 1.68 to 1.19 nmol/mg protein (by 29.2%). Untargeted lipidomics revealed that BSE normalized four lipid mediators linked to lipotoxicity, including CAR 22:1, LPC 16:0, PI‐Cer 38:1; O3, and NAE 26:5. Mechanistically, BSE activated AMPK signaling (increasing p‐AMPKα2 protein and *AMPKα2* mRNA expression by 1.72‐fold and 2.13‐fold, respectively) to inhibit lipogenesis (downregulating SREBP1 and SCD by 21.4% and 37.3%, respectively) and promote fatty acid oxidation (upregulating SIRT1, PPARα, CPT1A, and PGC1α by 1.3‐, 2.1‐, 4.6‐, and 1.3‐fold, respectively), enhanced Nrf2‐driven antioxidant defense (upregulating *Nrf2*, *HO‐1*, and *NQO1* mRNA expression by 1.2‐, 3.3‐, and 1.9‐fold, respectively), and suppressed the NLRP3 inflammasome cascade (reducing NLRP3 by 29.0%, cleaved caspase‐1 by 13.8%, and IL‐1β by 39.5%, respectively). These findings establish that BSE protects against HFD‐induced renal injury involving modulation of AMPK/Nrf2 signaling and the NLRP3 inflammasome, supporting its potential as a dietary strategy for metabolic kidney disease.

## Graphical abstract

High‐fat diet (HFD) induces renal lipid accumulation, AMPK suppression, oxidative stress, and NLRP3 inflammasome activation, leading to renal injury. Broccoli sprout extract (BSE) concurrently activates AMPK signaling (promoting fatty acid oxidation and inhibiting lipogenesis), enhances Nrf2‐mediated antioxidant defense, and suppresses NLRP3‐driven inflammation. Untargeted lipidomics reveals that BSE selectively normalizes four key lipid species (CAR 22:1, LPC 16:0, PI‐Cer 38:1, O3, and NAE 26:5) that bridge metabolic reprogramming and inflammation resolution. This coordinated multi‐target action breaks the vicious cycle of metabolic dysfunction, oxidative stress, and inflammation, thereby protecting against HFD‐induced renal injury.

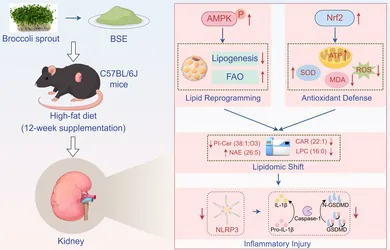

## 1 Introduction

The incidence and prevalence of chronic kidney disease (CKD) are increasing globally (Rao et al. 2023; Eisenstein 2023). CKD is a major contributor to morbidity and mortality worldwide, affecting over 10% of the global population, which translates to more than 800 million individuals (Francis et al. 2024). Numerous risk factors have been identified that may induce or exacerbate CKD, with hyperlipidemia, diabetes mellitus, and obesity being recognized as primary contributors (Fwu et al. 2024). In particular, chronic hyperlipidemia can lead to lipid accumulation in the kidney. This subsequently triggers lipotoxicity, which activates oxidative stress and pro‐inflammatory pathways, ultimately resulting in cellular injury and renal dysfunction (Suh and Kim 2023; Baek et al. 2022). Improving lipid profiles has been shown to be an effective strategy in decelerating the progression of CKD (Ferro et al. 2018). Several novel lipid‐targeting agents have been developed, and some are currently undergoing clinical trials in the context of CKD, fostering optimism for further therapeutic advancements in this area (Mitrofanova et al. 2023).

In the context of hyperlipidemia‐induced renal injury, AMP‐activated protein kinase (AMPK) serves as a pivotal upstream regulator (Qiu et al. 2023). Its suppressed activity under high‐fat diet (HFD) conditions not only promotes renal lipid deposition but also critically disrupts redox homeostasis (Rodríguez et al. 2020). This metabolic perturbation elevates oxidative stress, thus impairing the nuclear factor erythroid 2‐related factor 2 (Nrf2)‐mediated antioxidant defense system. AMPK directly counteracts this impairment by positively regulating Nrf2 signaling (Lu et al. 2023). Consequently, the unchecked oxidative stress further activates the NLRP3 inflammasome, creating a vicious cycle of inflammation and cellular damage that drives CKD progression (Islamuddin and Qin 2024). Therefore, therapeutic strategies capable of simultaneously activating AMPK and enhancing Nrf2 signaling may synergistically break this cycle, offering a potent approach to ameliorating lipid nephrotoxicity (Mitrofanova et al. 2023; Li et al. 2021).

Given the pivotal roles of AMPK/Nrf2 signaling suppression and NLRP3 inflammasome activation in HFD‐induced renal injury, dietary strategies capable of simultaneously modulating these pathways are of considerable interest. Broccoli sprout extract (BSE), a rich natural source of isothiocyanates, particularly sulforaphane (SFN), has garnered attention for its antioxidant, anti‐inflammatory, and nephroprotective properties (Vanduchova et al. 2019; Ramzan et al. 2025; Li, Wu, et al. 2025). While previous studies have demonstrated the protective effects of SFN in toxin‐ or obstruction‐induced kidney injury (Ramzan et al. 2025; Monteiro et al. 2023), these investigations have largely focused on purified compounds or single‐pathway readouts, leaving the efficacy and mechanism of whole BSE in a metabolic disorder setting largely unexplored.

The present study addresses this gap by employing whole BSE in a metabolic disorder model (HFD‐induced nephropathy), integrating untargeted lipidomics with pathway analysis to identify lipid species that may bridge metabolic reprogramming and inflammasome suppression, an approach not previously applied to BSE in kidney protection. Specifically, we aim to elucidate whether BSE exerts renoprotection through concurrent activation of the AMPK/Nrf2 axis and suppression of the NLRP3 inflammasome, and to provide a mechanistic foundation for its potential application as a dietary intervention in early CKD prevention.

## 2 Material and Methods

### 2.1 Chemicals and Reagents

Primary antibodies were obtained from the following commercial sources: antibodies against SCD, PGC1α, SIRT1, Fibronectin, TGF‐β1, SREBP1, IL‐6, TNF‐α, Nrf2, CPT1A, GAPDH, and β‐actin were from Bioworld Technology (MN, USA); antibodies against NLRP3, cleaved caspase‐1, and phospho‐AMPKα2 (Thr172) were from Affinity Biosciences (OH, USA); and the antibody against PPARα was from OriGene Technologies Inc. (MD, USA). Corresponding horseradish peroxidase (HRP)‐conjugated secondary antibodies, including goat anti‐rabbit IgG and goat anti‐mouse IgG, were purchased from Servicebio (Wuhan, China).

### 2.2 Animals and Diets

All animal experiments were approved by the Animal Care and Use Committee of Dali University (Permit No. SYXK [Dian] 2018‐0002). Six‐week‐old male C57BL/6J mice were procured from Kunming Chushang Technology Co. Ltd. (Kunming, Yunnan, China). Following a two‐week acclimatization period, the mice were fed either a low‐fat diet (LFD, 10% fat) or a high‐fat diet (HFD, 45% fat), with or without supplementation with broccoli sprout extract (BSE, 600 mg/kg diet), for 12 weeks (Li, Wu, et al. 2025). BSE (0.06% w/w) was homogeneously blended into the respective diets. At the start of the intervention, no significant differences in body weight were observed among the four groups (LFD: 17.63 ± 0.75 g; LFD + BSE: 17.49 ± 0.84 g; HFD: 17.58 ± 0.79 g; HFD + BSE: 17.60 ± 0.99 g; *p* > 0.05), confirming successful randomization. Body weight and food intake were recorded weekly. The detailed methods are provided in Supporting Methods.

### 2.3 BSE Preparation and Phytochemical Characterization

BSE was prepared from freeze‐dried broccoli sprouts ( *Brassica oleracea* L. var. *botrytis* L.) via ethanol extraction (70% aqueous ethanol, 1:20 w/v, 60°C, 2 h), concentration, and spray‐drying. The resulting powder was sieved, homogenized, vacuum‐packed in light‐protective bags, and stored at −20°C until use. The phytochemical composition of BSE was analyzed by ultra‐performance liquid chromatography‐high‐resolution mass spectrometry (UPLC‐HRMS). Detailed extraction procedures, chromatographic, and mass spectrometric conditions are provided in Supporting Methods.

### 2.4 Blood and Kidney Tissue Collection

After the 12‐week dietary intervention, mice were fasted for 8 h and anesthetized via an intraperitoneal injection of 10% chloral hydrate (0.02 mL/g body weight). Blood was collected via cardiac puncture into anticoagulant‐containing tubes. Plasma was obtained by sequential centrifugations (300× *g*, 2 min, 37°C; 500× *g*, 3 min, 37°C). The resulting supernatant was further centrifuged at 12,000× *g* for 10 min at 4°C to obtain platelet‐poor plasma and stored at −80°C. Following blood collection, mice were perfused with saline via the left ventricle. Kidneys were excised, weighed, and processed for further studies. The detailed methods are provided in Supporting Methods.

### 2.5 Histopathological Examination

For histological evaluation, harvested kidney tissues were fixed in 4% paraformaldehyde (PFA, pH 7.2), embedded in paraffin, and sectioned at a thickness of 5 μm. Hematoxylin and eosin (H&E) staining was then performed according to established protocols (Li, Zhang, et al. 2025; Cao et al. 2024) for general morphological assessment. Renal fibrosis was assessed using Masson's trichrome staining on deparaffinized sections. The staining procedure included sequential treatment with Weigert's hematoxylin, Masson blue, ponceau magenta, phosphomolybdic acid, and aniline blue (Guo et al. 2025; Zhang et al. 2025; Chen et al. 2025). Collagen fibers were stained blue, while cytoplasm and muscle fibers appeared red. The collagen volume fraction was calculated as the percentage of the blue‐stained area relative to the total tissue area in each field.

### 2.6 Biochemical Measurements

Serum creatinine (SCr), uric acid (UA), and blood urea nitrogen (BUN) levels were measured using an automatic biochemical analyzer (Beckman Instruments Inc., USA). Renal levels of total cholesterol (TC), triglycerides (TG), and free fatty acids (FFA) were determined using commercial kits. Oxidative stress markers, including total antioxidant capacity (T‐AOC), superoxide dismutase (SOD) activity, and malondialdehyde (MDA) content, were measured according to the manufacturers' instructions. Inflammatory cytokines TNF‐α and IL‐1β were quantified using specific ELISA kits. Absorbance readings and result calculations were performed according to the manufacturers' instructions. The detailed methods are provided in Supporting Methods.

### 2.7 Renal ATP Measurement

Renal ATP levels were determined using an ATP Assay Kit (Solarbio, Beijing, China) according to the manufacturer's protocol. Briefly, approximately 50 mg of kidney tissue was homogenized in lysis buffer and centrifuged at 12,000× *g* for 10 min at 4°C. The supernatant was collected for ATP measurement via a luciferase‐based chemiluminescent reaction, and luminescence intensity was recorded using a microplate reader. ATP concentrations were calculated from a standard curve and expressed as μmol/g tissue.

### 2.8 Immunohistochemical Analysis

Immunohistochemical staining was performed on paraffin‐embedded kidney sections to evaluate the expression of specific proteins. The sections were incubated with primary antibodies against the following targets: TNF‐α (1:100), nuclear factor erythroid 2‐related factor 2 (Nrf2; 1:100), IL‐1β (1:100), and NLRP3 (1:100). Following appropriate secondary antibody incubation and color development, all stained sections were visualized and digitized using an Olympus microscope (DP74, Tokyo, Japan). Protein expression levels were quantified based on the integrated optical density (IOD) of positive staining using Image Pro Plus 6.0 software (Media Cybernetics, USA).

### 2.9 Quantitative Reverse Transcription Polymerase Chain Reaction (qRT‐PCR)

Total RNA was extracted from kidney tissues using an Animal Tissue Total RNA Extraction Kit (Servicebio, Wuhan, China). Subsequently, 1 μg of total RNA was reverse‐transcribed into complementary DNA (cDNA) using the SweScript All‐in‐One RT SuperMix for qPCR (Servicebio). qRT‐PCR was performed with the SuperReal PreMix Plus (TianGen Biotech Co. Ltd., Beijing, China) on a QuantStudio 3 Real‐Time PCR System (Thermo Fisher Scientific, MA, USA). The primer sequences for target genes (*AMPKα2*, heme oxygenase‐1 (HO‐1), NAD(P)H quinone oxidoreductase 1 (*NQO1*), *NLRP3*, and *Nrf2*) and the reference gene *GAPDH* are listed in Table S1. The relative mRNA expression levels of the target genes were normalized to *GAPDH* and calculated using the 2^^–ΔΔCt^ method.

### 2.10 Western Blot Analysis

Kidney tissues were homogenized in RIPA lysis buffer containing protease and phosphatase inhibitors (Beyotime Biotechnology, Shanghai, China). Proteins were separated by SDS‐PAGE and transferred onto PVDF membranes (Millipore, USA) (Li, Wu, et al. 2025; Ma et al. 2026; Zhou et al. 2023). After blocking with 5% BSA for 1.5 h, the membranes were cut into strips based on the molecular weight of the target proteins, and each strip was incubated overnight at 4°C with its corresponding primary antibody. Primary antibodies against the following proteins were used: Fibronectin, TGF‐β1, SREBP1, SCD, SIRT1, PPARα, CPT1A, PGC1α, phospho‐AMPKα2 (Thr172), Nrf2, TNF‐α, IL‐6, NLRP3, cleaved caspase‐1, GAPDH, and β‐actin. The membrane strips were not subjected to stripping and reprobing; each target protein and its corresponding loading control were obtained from the same membrane. Following incubation with appropriate HRP‐conjugated secondary antibodies, bands were visualized using an ECL detection system. Band intensities were quantified with ImageJ software (version 1.37v; NIH, USA) and normalized to the corresponding loading control. Loading controls (GAPDH or β‐actin) were selected based on molecular weight compatibility with each target protein to avoid signal overlap. Both housekeeping proteins were confirmed to be stably expressed across all experimental groups. The detailed methods are provided in Supporting Methods.

### 2.11 Untargeted Lipidomics Analysis

Kidney lipids were extracted using MTBE‐methanol, and untargeted lipidomics was performed using UPLC‐TOF/MS (TripleTOF 6600, SCIEX). Data were processed by XCMS; metabolites were annotated against KEGG and HMDB databases. Lipids with VIP > 1.0 and FDR‐adjusted *p* < 0.05 were considered significantly altered. The detailed methods are provided in Supporting Methods.

### 2.12 Statistical Analysis

All data were obtained from at least three independent experiments and are expressed as the means with standard deviation (mean ± SD). For Western blotting, immunohistochemistry, and histological analyses, three randomly selected mice per group were used as biological replicates. Statistical comparisons among groups were performed by one‐way analysis of variance (ANOVA) followed by Tukey's post hoc test using GraphPad Prism software (version 9.4.1; GraphPad Inc., San Diego, CA, USA). Differences between groups were considered statistically significant at *p* value below 0.05.

## 3 Results

### 3.1 Phytochemical Composition of BSE

**FIGURE 1:**
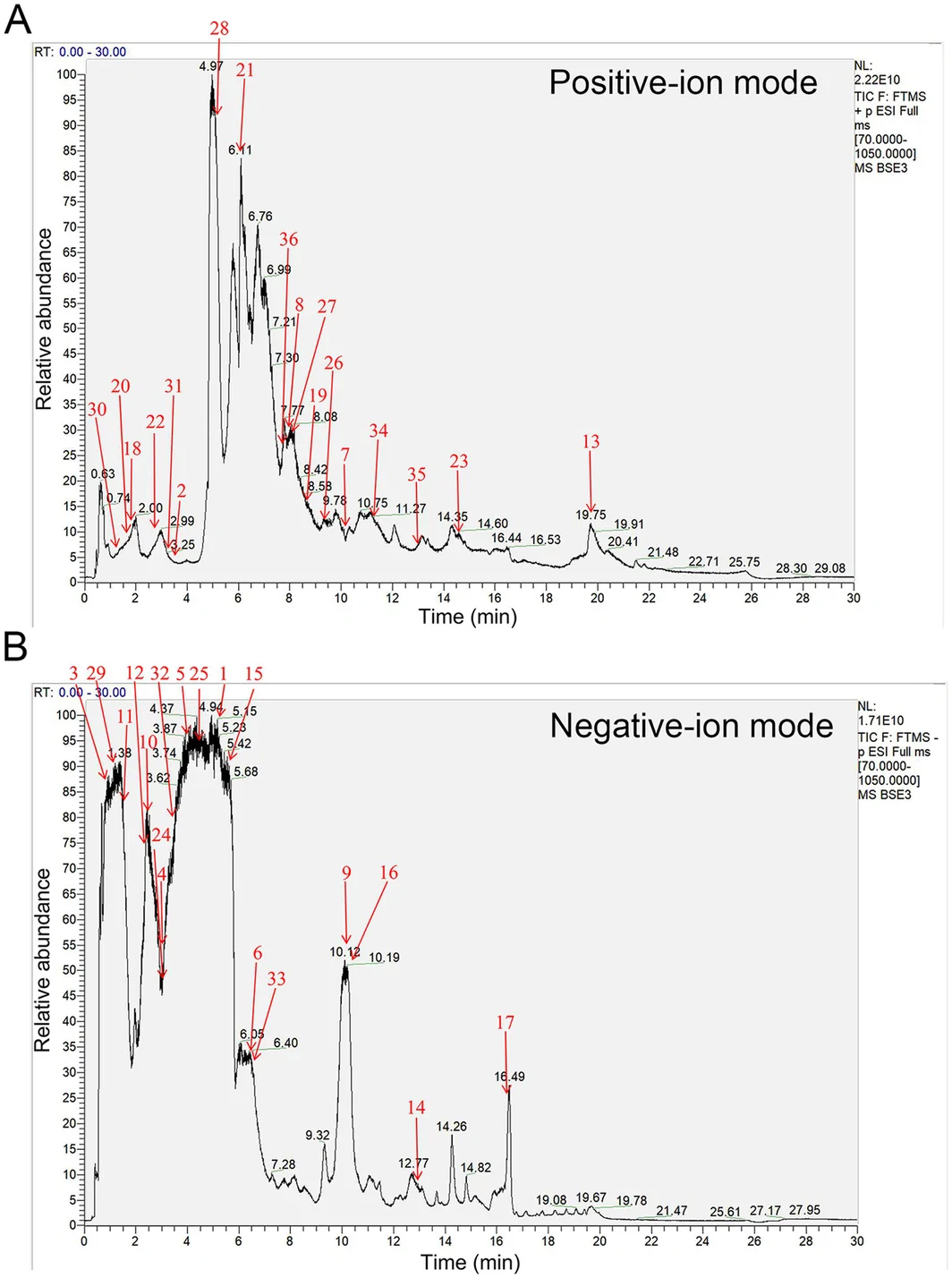
Phytochemical composition of BSE. Total ion chromatograms of BSE. (A) Positive‐ion mode. (B) Negative‐ion mode. Peaks corresponding to the major metabolites identified in Table 1 are labeled with their respective numbers, representing key compound classes such as carbohydrates, isothiocyanates, hydroxycinnamic acids, and amino acid derivatives.

The UPLC‐HRMS analysis revealed a diverse chemical profile. Representative base peak intensity (BPI) chromatograms in positive‐ and negative‐ion modes were shown in Figure 1A,B, respectively. Compounds were annotated at Level 2 (putatively identified) according to the Metabolomics Standards Initiative (MSI), based on accurate mass, retention time, and fragmentation patterns matching public databases (HMDB, KEGG) and literature. Among the 36 major annotated peaks (Table 1), the most abundant classes included carbohydrates and their conjugates (e.g., glucoraphanin, sinigrin, stachyose), hydroxycinnamic acids (e.g., sinapine), and isothiocyanates. Other classes such as amino acids, organic acids, and nitrogen‐containing compounds were also identified. Retention times ranged from 43.3 to 1191.9 s, reflecting broad polarity diversity among the constituents. Quantitative analysis of BSE, performed as we previously described (Zhou et al. 2023), showed that it contained approximately 72% total glucosinolates, 10% SFN, and 12% total phenolics. Notably, glucosinolates, including glucoraphanin and sinigrin, are carbohydrate conjugates, consistent with their prominent detection in the UPLC‐HRMS profile.

**TABLE 1:** Phytochemical profiling of BSE: Major constituents identified by UPLC‐HRMS.

No.	Metabolite name	Subclass	Formula	Adducted Ion	*m*/*z*	Retention time (s)	MSI Level
1	Glucoraphanin	Carbohydrates and carbohydrate conjugates	C_12_ H_23_NO_10_S_3_	[M‐H]^−^	436.038	299.405	Level 1*
2	Sinapine	Hydroxycinnamic acids and derivatives	C_16_H_24_NO_5_	[M]^+^	310.162	372.222	Level 1*
3	Glucoerusin	Carbohydrates and carbohydrate conjugates	C_12_H_23_NO_9_S_3_	[M‐H]^−^	420.043	68.446	Level 1*
4	N‐[4‐(2‐Chlorophenyl)‐1,3‐Dioxo‐1,2,3,6‐Tetrahydropyrrolo[3,4‐C]carbazol‐9‐Yl]formamide	Carbazoles	C_21_H_12_ClN_3_O_3_	[M‐H]^−^	388.035	151.125	Level 2^a^
5	Glucoraphanin (4‐Methylsulfinylbutyl‐GS)	Carbohydrates and carbohydrate conjugates	C_12_H_23_NO_10_S_3_	[M‐H]^−^	436.038	241.023	Level 1*
6	Ascorbic acid	Furanones	C_6_H_8_O_6_	[M‐H]^−^	175.023	374.386	Level 1*
7	Choline	Quaternary ammonium salts	C_5_H_14_NO	[M]^+^	104.106	653.279	Level 1*
8	4,7‐dimethoxy‐1‐methyl‐N‐(propan‐2‐yl)‐1H‐indole‐2‐carboxamide	Indolecarboxylic acids and derivatives	C_15_H_20_N_2_O_3_	[M + H]^+^	277.153	477.481	Level 2^a^
9	Trehalose	Carbohydrates and carbohydrate conjugates	C_12_H_22_O_11_	[M‐H]^−^	341.106	609.042	Level 1*
10	DYAQCRHEYVANDL‐HOQQJHGQSA‐M	Carbohydrates and carbohydrate conjugates	C11H20NO10S2—	[M‐H]^−^	389.037	151.125	Level 2^a^
11	Sinigrin	Carbohydrates and carbohydrate conjugates	C_10_H_17_NO_9_S_2_	[M‐H]^−^	358.024	93.613	Level 1*
12	methyl‐oxido‐[(6E)‐6‐sulfooxyimino‐6‐[(2S,3R,4S,5S,6R)‐3,4,5‐trihydroxy‐6‐(hydroxymethyl)oxan‐2‐yl]sulfanylhexyl]sulfanium	Carbohydrates and carbohydrate conjugates	C_13_H_25_NO_10_S_3_	[M‐H]^−^	450.053	194.959	Level 2^a^
13	L‐Arginine	Amino acids, peptides, and analogues	C_6_H_14_N_4_O_2_	[M + H]^+^	175.118	1191.919	Level 1*
14	Malicacid	Beta hydroxy acids and derivatives	C_4_H_6_O_5_	[M‐H]^−^	133.013	770.232	Level 1*
15	Glucoiberin	Carbohydrates and carbohydrate conjugates	C_11_H_21_NO_10_S_3_	[M‐H]^−^	422.023	337.676	Level 1*
16	Sucrose	Carbohydrates and carbohydrate conjugates	C_12_H_22_O_11_	[M + C2H3O2]^−^	401.127	609.600	Level 1*
17	Stachyose	Carbohydrates and carbohydrate conjugates	C_24_H_42_O_21_	[M‐H]^−^	665.210	984.225	Level 1*
18	4‐hydroxycinnamamide	Hydroxycinnamic acids and derivatives	C_9_H_9_NO_2_	[M + H]^+^	164.073	112.833	Level 2^a^
19	(1‐(sec‐butyl)‐1H‐indol‐3‐yl)(naphthalen‐2‐yl)‐methanone	Benzoylindoles	C_23_H_21_NO	[M + H]^+^	328.173	508.258	Level 2^a^
20	1,3‐Diiminoisoindoline	Isoindoles	C_8_H_7_N_3_	[M + H]^+^	146.062	43.299	Level 2^a^

21	3‐morpholin‐4‐yl‐5H‐pyrimido[4,5‐e][1,2,4]triazine‐6,8‐dione	Amines	C_9_H_10_N_6_O_3_	[M + H]^+^	251.090	371.986	Level 2^a^
22	(2S,3R,4S,5R)‐2‐(6‐aminopurin‐9‐yl)‐5‐(hydroxymethyl)oxolane‐3,4‐diol	Purine nucleosides	C_10_H_13_N_5_O_4_	[M + H]^+^	268.102	178.488	Level 2^a^
23	14‐cycloheptyl‐5,6‐dihydroxy‐10‐ (4‐hydroxy‐3,5‐dimethoxyphenyl)‐8‐oxa‐13,14,16‐triazatetracyclo [7,7,0.02,7.0,11,15] hexadeca‐1(16),2,4,6,9,11(15)‐hexaen‐12‐one	Methoxyphenols	C_27_H_27_N_3_O_7_	[M + H]^+^	506.201	878.676	Level 2^a^
24	(10S)‐10‐(3‐chloro‐4‐hydroxyphenyl)‐5‐hydroxy‐3‐(4‐methoxyphenyl)‐9,10‐dihydropyrano[2,3‐h]chromene‐4,8‐dione	Pyranoisoflavonoids	C_25_H_17_ClO_7_	[M‐H]^−^	463.045	181.402	Level 2^a^
25	Glucoalyssin	Carbohydrates and carbohydrate conjugates	C_13_H_25_NO_10_S_3_	[M‐H]^−^	450.053	254.714	Level 1*
26	N‐benzyl‐1‐[(2‐fluorophenyl)methyl]pyridin‐4‐imine	Halobenzenes	C_19_FN_2_	[M + H]^+^	293.147	576.412	Level 2^a^
27	4‐methoxy‐5‐[5‐[(4‐methoxybenzoyl)amino]‐1‐methylbenzimidazol‐2‐yl]‐1‐methyl‐N‐propan‐2‐ylpyrrole‐3‐carboxamide	Benzimidazoles	C_26_H_29_N_5_O_4_	[M + H]^+^	476.224	477.761	Level 2^a^
28	2‐(2‐hydroxyethyl)‐7,8‐dimethoxyphthalazin‐1 (2H)‐one	Benzodiazines	C_12_H_14_N_2_O_4_	[M + H]^+^	251.090	302.581	Level 2^a^
29	5‐chloro‐13‐methyl‐3,13,23‐triazahexacyclo [14,7,0.02,10.04,9.0,11,15.0,17,22] tricosa‐1,4(9),5,7,10,15,17,19,21‐nonaene‐12,14‐dione	Carbazoles	C_21_H_12_ClN_3_O_2_	[M‐H]^−^	372.040	81.325	Level 2^a^
30	Sulforaphane	—	C_6_H_11_NOS_2_	[M + H]^+^	178.034	75.391	Level 1*
31	Sulforaphane‐cysteine‐glycine	Amino acids, peptides, and analogues	C_11_H_21_N_3_O_4_S_3_	[M + H]^+^	356.063	224.775	Level 1*
32	1‐Isothiocyanato‐6‐(methylthio)hexane	—	C_8_H_15_NS_2_	[M‐H]^−^	188.055	186.073	Level 2^a^
33	hydroxy‐(4‐isothiocyanatobutyl)‐methyl‐λ^4^‐sulfane	Isothiocyanates	C_6_H_13_NOS_2_	[M‐H]^−^	178.031	375.384	Level 2^a^
34	Unidentified metabolite	Isothiocyanates	C_9_H_18_NOS_2_	[M + H]^+^	221.094	668.548	Level 3^b^
35	(R)‐8‐Methylsulfinyloctyl isothiocyanate	Isothiocyanates	C_10_H_19_NOS_2_	[M + H]^+^	234.096	779.850	Level 2^a^
36	4‐methylsulfinyl butyl isothiocyanate	—	C_6_H_11_NOS_2_	[M + H]^+^	178.034	456.813	Level 2^a^

* MSI Level 1: Confirmed by comparison with authentic standards.

^a^ MSI Level 2: Putatively identified based on accurate mass, retention time, and fragmentation patterns matching public databases (HMDB, KEGG) and literature.

^b^ MSI Level 3: Tentatively assigned based on accurate mass only; no MS/MS or database match available.

### 3.2 BSE Ameliorates HFD‐Induced Renal Injury and Dyslipidemia

**FIGURE 2:**
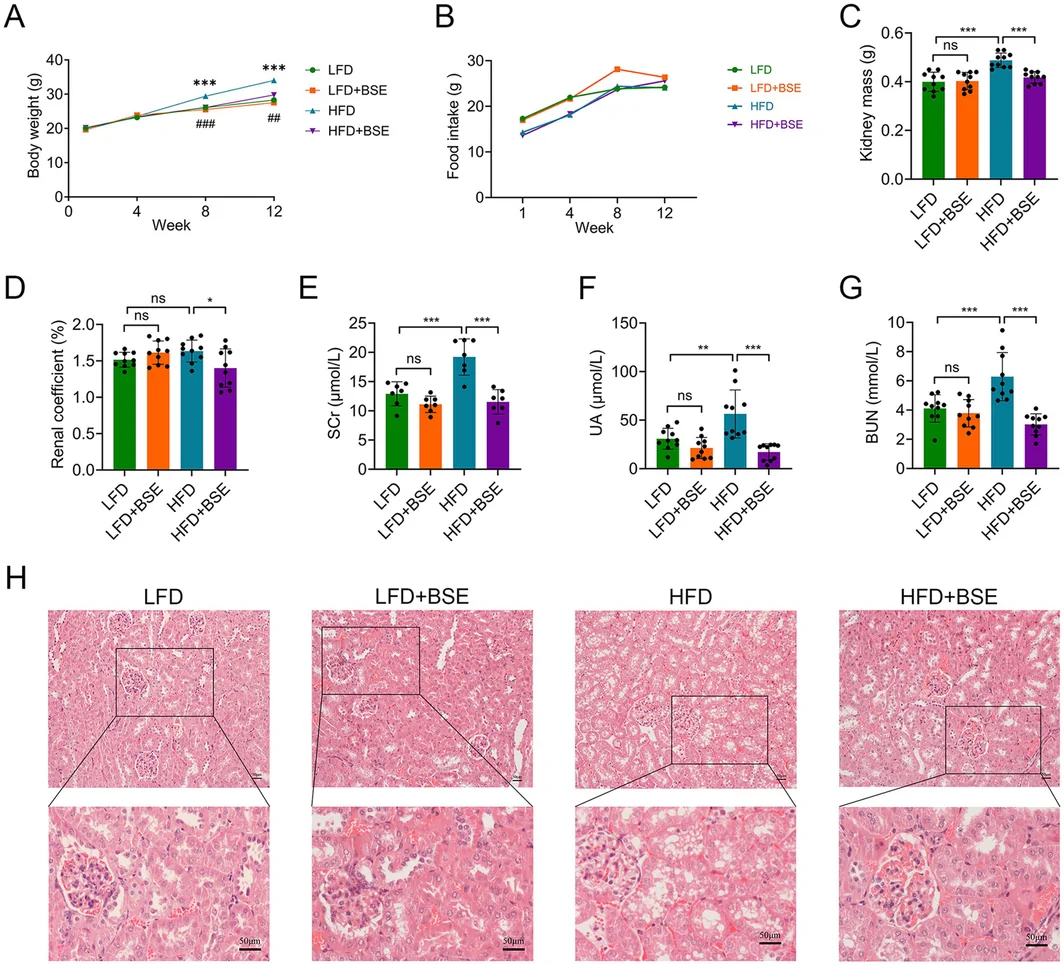
BSE ameliorates HFD‐induced renal injury and dyslipidemia. (A, B) Body weight (A) and food intake (B) were monitored in the LFD, LFD + BSE, HFD, and HFD + BSE groups. ***p < 0.001: LFD group vs. HFD group; ^##^ *p* < 0.01, ^###^ *p* < 0.001: HFD group vs. HFD + BSE group (*n* = 10). (C) Kidney weight was measured at the end of the 12 week intervention (*n* = 10). (D) Renal coefficient was calculated as kidney weight/body weight (*n* = 10). (E–G) Levels of SCr (E), serum UA (F), and BUN (G) were determined (*n* = 7–10). (H) Representative H&E staining images of kidney sections (*n* = 3). Scale bar = 50 μm. *p < 0.05, **p < 0.01, and ***p < 0.001; ns, not significant.

As shown in Figure 2A, body weight in the HFD group became significantly higher than in the LFD group from the 8th week onward; this increase was attenuated by BSE supplementation. No significant differences in food intake were observed among the groups during the study (Figure 2B). After 12 weeks, renal weight was significantly greater in the HFD group than in the LFD group, but was reduced in HFD + BSE mice (Figure 2C). Although the kidney‐to‐body weight ratio did not differ significantly between the HFD and LFD groups, a trend toward increase was observed in HFD‐fed mice, which tended to be reversed by BSE supplementation (Figure 2D). Additionally, BSE supplementation favorably improved the plasma lipid profile in HFD‐fed mice, reducing TC, TG, and low‐density lipoprotein cholesterol (LDL‐C), while increasing high‐density lipoprotein cholesterol (HDL‐C) (Li, Wu, et al. 2025).

To assess renal function, we measured key biochemical markers. Levels of SCr, UA, and BUN were significantly elevated in the HFD group compared with the LFD group, and these changes were significantly ameliorated by BSE supplementation (Figure 2E–G). Furthermore, histopathological analysis via H&E staining revealed normal renal morphology in the LFD and LFD + BSE groups. In contrast, kidney sections from HFD‐fed mice exhibited substantial injury, including irregular glomerular contours, structural disintegration of Bowman's capsule, vacuolar degeneration of tubular epithelial cells, severe brush border loss, and epithelial sloughing. These pathological features were notably alleviated in the HFD + BSE group (Figure 2H). Taken together, these results indicate that BSE effectively attenuates HFD‐induced renal injury and improves associated metabolic and histopathological alterations in mice.

### 3.3 BSE Attenuates Renal Lipid Accumulation and Fibrosis

**FIGURE 3:**
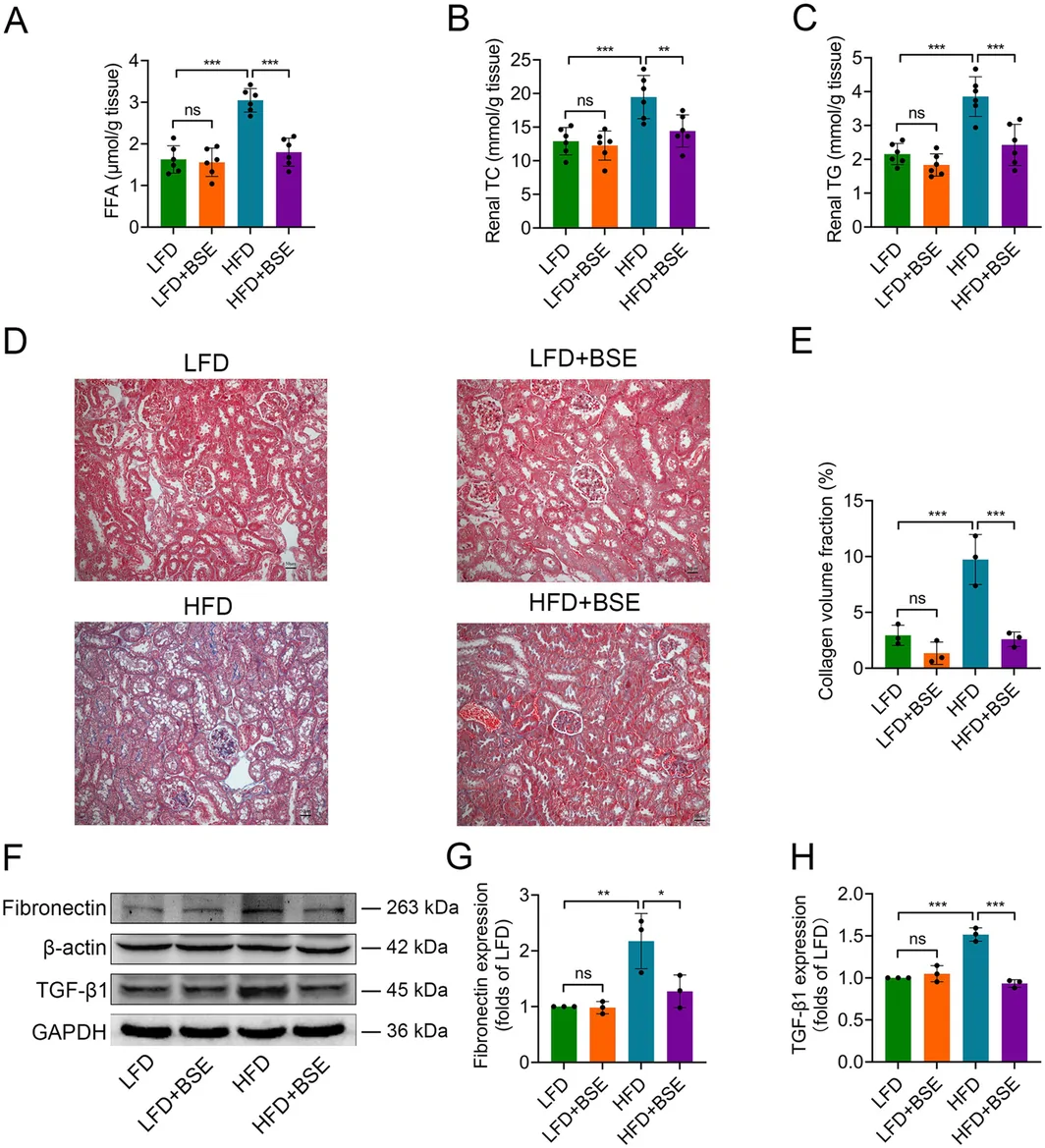
BSE attenuates renal lipid accumulation and fibrosis. (A–C) Renal levels of FFA (A), TC (B), and TG (C) were measured (*n* = 6). (D, E) Representative Masson's trichrome staining images of kidney sections (D) and quantitative analysis of the collagen volume fraction (E) (*n* = 3). The collagen volume fraction was calculated as the percentage of blue‐stained (collagen‐positive) area relative to the total tissue area per field. Scale bar = 50 μm. (F–H) Protein expression of fibrotic markers was assessed by Western blot. (F) Representative immunoblots of fibronectin and TGF‐β1. (G, H) Densitometric analysis of fibronectin (G) and TGF‐β1 (H) expression normalized to β‐Actin (fibronectin) or GAPDH (TGF‐β1) (*n* = 3). *p < 0.05, **p < 0.01 and ***p < 0.001; ns, not significant.

HFD feeding significantly increased renal lipid content, as evidenced by elevated levels of FFA, TC, and TG compared with the LFD group. BSE supplementation effectively attenuated these HFD‐induced increases, indicating its capacity to mitigate renal lipid accumulation (Figure 3A–C). Concurrently, renal fibrosis was assessed by Masson's trichrome staining, which revealed a marked increase in collagen fiber deposition in the kidneys of HFD‐fed mice. This fibrotic response was significantly suppressed by BSE treatment (Figure 3D,E). Consistent with the histological findings, the expression levels of key fibrotic markers were analyzed. Western blot analysis showed that HFD feeding significantly upregulated the protein expression of fibronectin and TGF‐β1, and these changes were reversed by BSE supplementation (Figure 3F–H). Taken together, these results demonstrate that BSE effectively prevents HFD‐induced renal lipid accumulation and fibrosis.

### 3.4 Untargeted Lipidomics Reveals Remodeling of Renal Lipid Metabolism by BSE

To investigate the alterations in renal lipid profiles, an untargeted lipidomics analysis was performed using UPLC‐TOF/MS. Following data preprocessing and filtering (RSD < 30%), a total of 2069 lipid compounds were annotated across all kidney samples, comprising 1668 and 401 compounds detected in ESI^+^ and ESI^−^ modes, respectively. These lipids were categorized into 82 subclasses. Phosphatidylcholines (PC, 23.30%), triglycerides (TG, 8.65%), sphingomyelins (SM, 7.20%), cardiolipins (CL, 6.43%), and diacylglycerols (DG, 5.12%) represented the most abundant subclasses (see distribution in Figure S1). Representative and reproducible base peak intensity (BPI) chromatograms for both ionization modes are provided in Figure S2.

**FIGURE 4:**
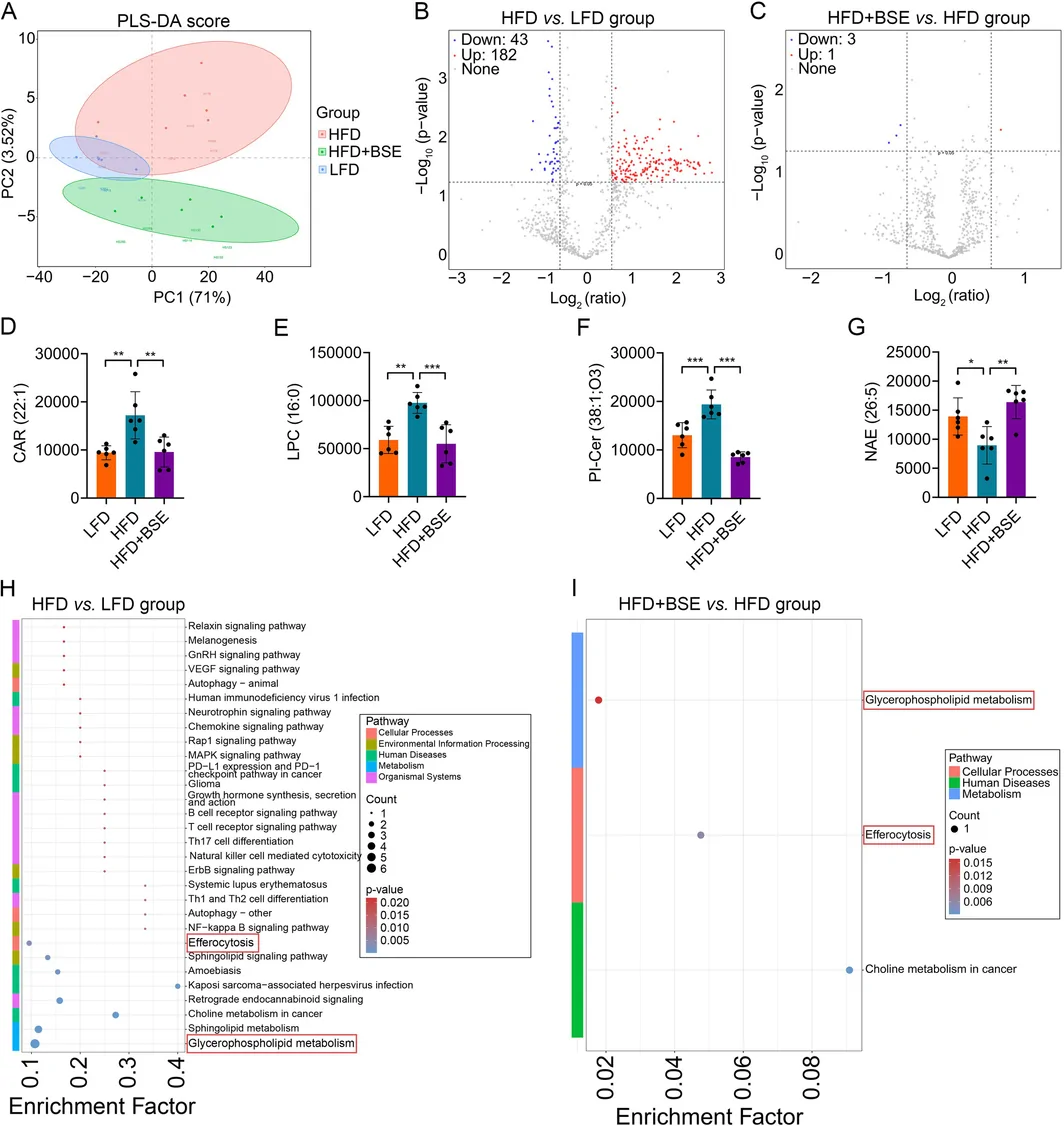
Untargeted lipidomics reveals remodeling of renal lipid metabolism by BSE (A) PLS‐DA score plot showing the separation among the LFD, HFD, and HFD + BSE groups. (B, C) Volcano plots of differentially abundant lipids in the LFD versus HFD comparison (B) and the HFD versus HFD + BSE comparison (C). (D–G) Relative levels of the significantly altered lipids CAR (22:1) (D), LPC (16:0) (E), PI‐Cer (38:1; O3) (F), and NAE (26:5) (G) based on peak area quantification. (H, I) KEGG pathway enrichment analysis of differential metabolites for the HFD versus LFD comparison (H) and the HFD + BSE versus HFD comparison (I). *p < 0.05, **p < 0.01, and ***p < 0.001.

To characterize the global metabolic shifts among the experimental groups, partial least squares‐discriminant analysis (PLS‐DA) was employed. The model effectively captured 74.52% of the total variance in the first two principal components. The score plot demonstrated a pronounced separation between the LFD and HFD groups along the first component, whereas the HFD and HFD + BSE groups exhibited a more subtle, yet discernible, separation (Figure 4A). Differential lipids were identified based on a variable importance in projection (VIP) score > 1.0 and an FDR‐corrected *p* value < 0.05. Hierarchical clustering of these lipids revealed distinct profiles across the three groups. Comparative analysis showed that HFD feeding induced substantial lipidomic dysregulation, with 182 lipids significantly upregulated and 43 downregulated relative to the LFD group (Figure 4B). In striking contrast, BSE intervention elicited a highly targeted effect, significantly modulating only 4 lipids relative to the HFD group (3 downregulated, 1 upregulated) (Figure 4C). Specifically, BSE treatment attenuated the HFD‐induced increases in acylcarnitine (CAR 22:1), lysophosphatidylcholine (LPC 16:0), and phosphatidylinositol ceramide (PI‐Cer 38:1; O3), while elevating the HFD‐suppressed level of N‐acylethanolamine (NAE 26:5) (Figure 4D–G). Pathway enrichment analysis of the differential metabolites highlighted significant perturbations in glycerophospholipid metabolism and efferocytosis pathways in HFD‐fed mice. Importantly, BSE supplementation was found to specifically counteract these metabolic disturbances (Figure 4H,I).

### 3.5 BSE Activates AMPK Signaling to Modulate Lipid Metabolism

**FIGURE 5:**
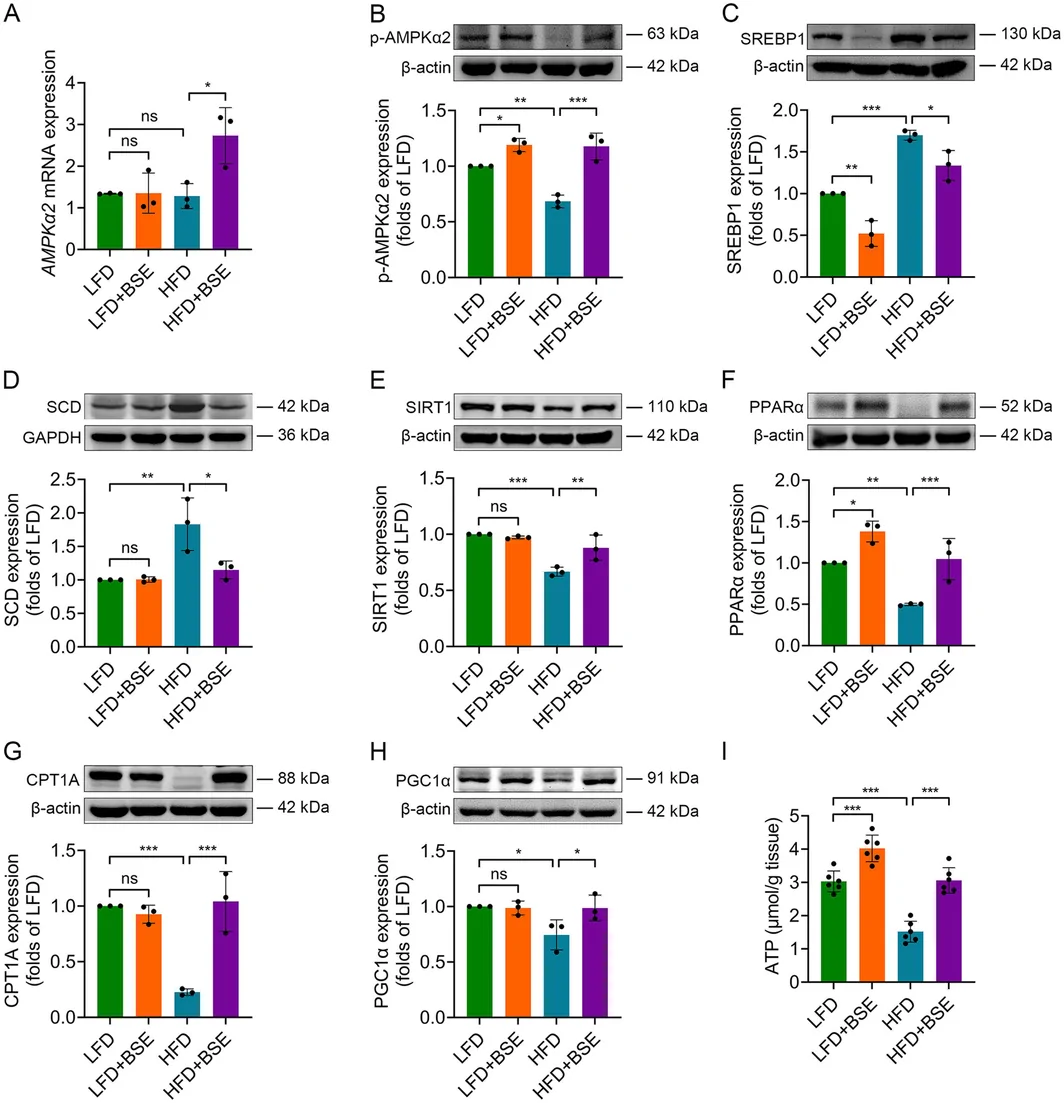
BSE activates AMPK signaling to modulate lipid metabolism. (A) *AMPKα2* mRNA expression levels (*n* = 3). (B) Protein levels of phosphorylated AMPKα2 (Thr172) analyzed by Western blot. (C–H) Western blot analysis of proteins involved in lipogenesis and fatty acid oxidation: SREBP1 (C), SCD (D), SIRT1 (E), PPARα (F), CPT1A (G), and PGC1α (H) (*n* = 3). (I) Renal ATP levels (*n* = 6). *p < 0.05, **p < 0.01, and ***p < 0.001; ns, not significant.

Given this specific lipidomic reprogramming, we focused on the AMPK pathway to elucidate the underlying metabolic mechanism (Li et al. 2021). AMPK exists in two catalytic isoforms (α1 and α2), of which the α2 isoform is predominantly expressed in the kidney and plays a dominant role in regulating lipid metabolism and mitochondrial function (Steinberg and Kemp 2009; Li et al. 2020). We therefore examined AMPKα2 as the representative catalytic subunit in this study. At the transcriptional level, *AMPKα2* mRNA expression was significantly increased specifically in the HFD + BSE group (Figure 5A), suggesting that BSE may enhance the expression of the *AMPKα2* gene in the context of lipid metabolic disturbance. Western blot analysis also revealed that the phosphorylation of AMPKα2 at Thr172, indicative of its activation, was markedly suppressed in the kidneys of HFD‐fed mice compared to the LFD group (Figure 5B). BSE supplementation significantly restored AMPK phosphorylation in both HFD‐ and LFD‐fed mice, indicating its potent activating effect on this pathway, particularly under metabolic stress (Figure 5B).

We next examined key downstream effectors of AMPK involved in lipid synthesis. Western blot analysis revealed that the HFD significantly increased the protein levels of SREBP1 and its target gene SCD (Figure 5C,D), both critical promoters of lipogenesis. BSE supplementation effectively reversed these increases (Figure 5C,D). In LFD‐fed mice, BSE also reduced SREBP1 but not SCD, suggesting a primary effect on suppressing *de novo* lipogenic pathways (Figure 5C,D). Additionally, we evaluated proteins involved in fatty acid oxidation (FAO). The expression of SIRT1, PPARα, CPT1A, and PGC1α—key regulators of the FAO pathway (Tan et al. 2023)—was significantly downregulated in the HFD group. BSE intervention robustly increased the expression of these proteins (Figure 5E–H). Consistent with enhanced mitochondrial FAO, BSE also restored the HFD‐reduced renal ATP levels (Figure 5I). Collectively, these findings demonstrate that BSE modulates renal lipid metabolism by activating AMPK, which in turn suppresses lipogenesis and promotes fatty acid oxidation, ultimately improving cellular energy homeostasis.

### 3.6 BSE Activates the Nrf2 Pathway to Alleviate Oxidative Stress

**FIGURE 6:**
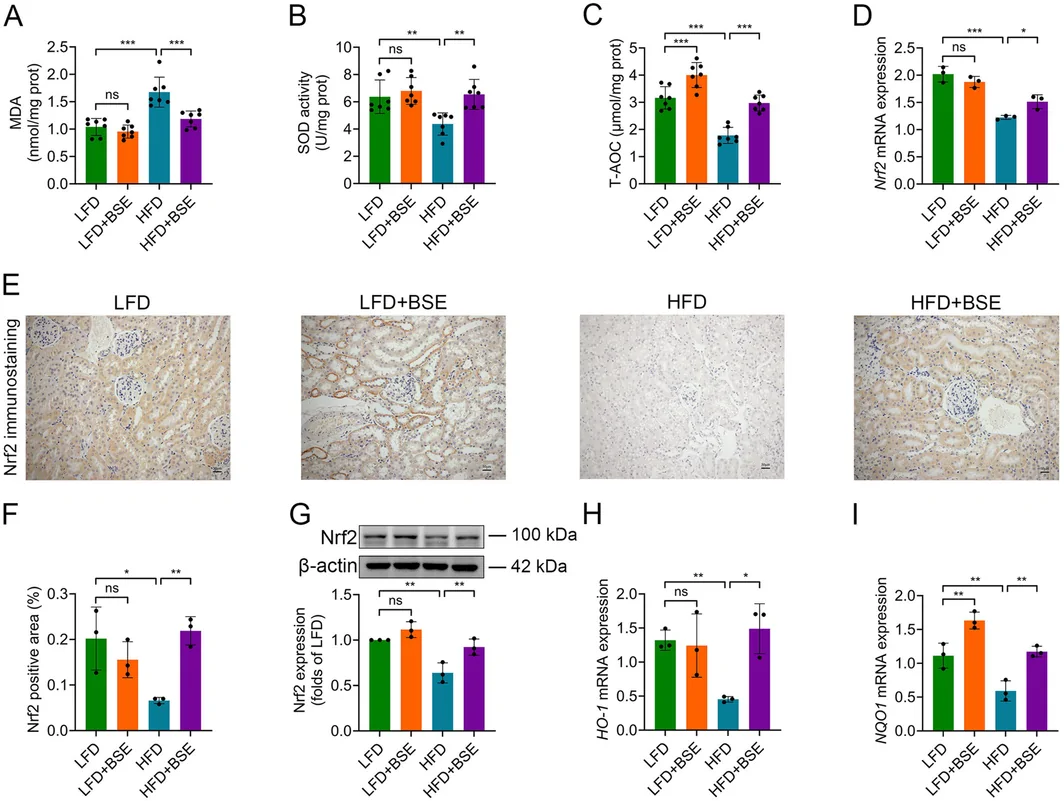
BSE activates the Nrf2 pathway to alleviate oxidative stress. (A–C) Renal levels of MDA (A), SOD activity (B), and T‐AOC (C) (*n* = 7). (D) *Nrf2* mRNA expression (*n* = 3). (E–F) Representative immunohistochemical staining images of Nrf2 (E) and quantitative analysis of Nrf2‐positive area (F) (*n* = 3). Scale bar = 50 μm. (G) Western blot analysis of Nrf2 protein expression (*n* = 3). (H, I) mRNA expression levels of the Nrf2 downstream genes *HO‐1* (H) and *NQO1* (I) (*n* = 3). *p < 0.05, **p < 0.01, and ***p < 0.001; ns, not significant.

Initial assessment of redox status in renal tissues showed that HFD feeding induced significant oxidative stress, marked by an increase in MDA alongside decreases in SOD activity and T‐AOC. BSE supplementation effectively ameliorated all these alterations (Figure 6A–C), demonstrating its capacity to restore renal antioxidant defenses under HFD conditions.

We next investigated the Nrf2 pathway, a central transcriptional regulator of antioxidant responses (Liu et al. 2022). Consistent across qRT‐PCR (mRNA), Western blot (total protein), and immunohistochemistry (tissue localization) analyses, Nrf2 expression was significantly downregulated in the kidneys of HFD‐fed mice. BSE intervention notably reversed this suppression, enhancing Nrf2 levels across all measured dimensions (Figure 6D–G). Activated Nrf2 translocates to the nucleus and drives the expression of cytoprotective genes through the antioxidant response element (ARE) (Hassanein et al. 2023). Accordingly, we examined the transcription of two key downstream targets, HO‐1 and NQO1. HFD feeding substantially suppressed their mRNA expression, while BSE treatment robustly restored the transcript levels of both genes (Figure 6H,I). In summary, these findings indicate that BSE alleviates HFD‐induced renal oxidative stress by activating the Nrf2 signaling pathway and subsequently enhancing the expression of its downstream antioxidant genes.

### 3.7 BSE Suppresses NLRP3 Inflammasome Activation and Renal Inflammation

**FIGURE 7:**
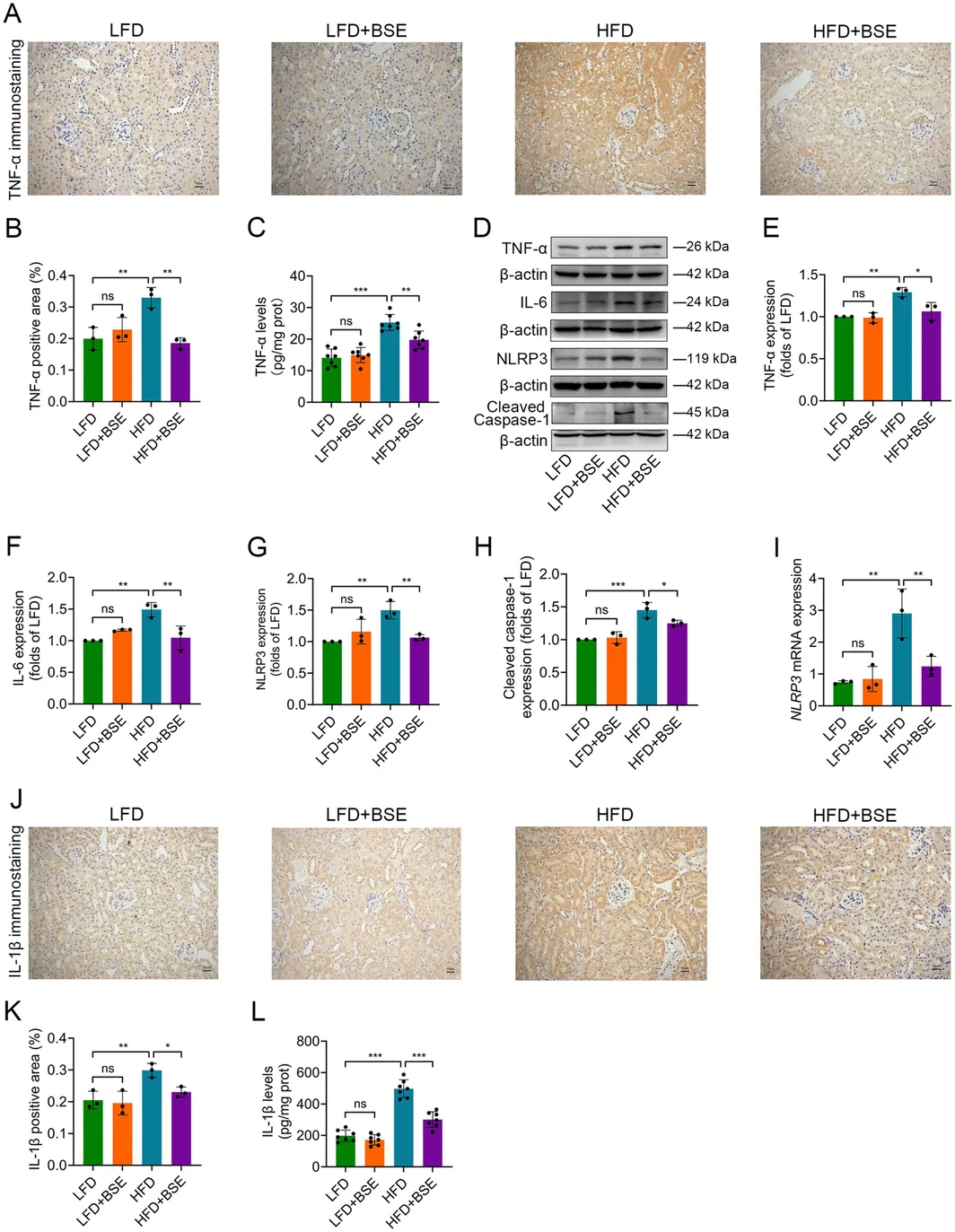
BSE suppresses NLRP3 inflammasome activation and renal inflammation. (A–C) Analysis of renal TNF‐α expression. Representative immunohistochemical staining images (A) (Scale bar = 50 μm), quantitative analysis of the TNF‐α‐positive area (B), and ELISA measurement of TNF‐α protein levels (C) (*n* = 3 for (A, B); *n* = 7 for (C)). (D–H) Western blot analysis of inflammatory mediators. (D) Representative immunoblots of TNF‐α, IL‐6, NLRP3, and cleaved caspase‐1. (E–H) Densitometric quantification of TNF‐α (E), IL‐6 (F), NLRP3 (G), and cleaved caspase‐1 (H) expression, normalized to β‐Actin (*n* = 3). (I) *NLRP3* mRNA expression levels (*n* = 3). (J–L) Analysis of mature IL‐1β. Representative immunohistochemical staining images (J) (Scale bar = 50 μm), quantitative analysis of the IL‐1β‐positive area (K), and ELISA measurement of IL‐1β levels (L) (*n* = 3 for (J, K); *n* = 7 for (L)). *p < 0.05, **p < 0.01, and ***p < 0.001; ns, not significant.

Chronic inflammation synergizes with dyslipidemia to promote renal injury. To evaluate the effect of BSE on renal inflammation, we first measured general pro‐inflammatory markers. HFD feeding significantly elevated the expression of key cytokines. Specifically, immunohistochemical staining revealed increased TNF‐α deposition in renal tissue (Figure 7A,B), which was corroborated by elevated TNF‐α levels in both ELISA (Figure 7C) and Western blot analyses (Figure 7D,E). Similarly, Western blot showed a marked upregulation of IL‐6 protein in the HFD group (Figure 7D,F). BSE supplementation significantly attenuated the HFD‐induced increase in both TNF‐α and IL‐6 across all detection methods (Figure 7A–F), demonstrating its broad anti‐inflammatory effect.

Given the central role of the NLRP3 inflammasome in amplifying inflammatory responses (Islamuddin and Qin 2024), we next investigated its activation status. HFD feeding led to significant activation of this pathway, evidenced by increased NLRP3 expression at both the protein (Figure 7D,G) and mRNA (Figure 7I) levels. This activation was functionally consequential, as indicated by the elevated protein level of cleaved caspase‐1 (Figure 7D,H), the active enzyme that processes pro‐IL‐1β (Sharma and Kanneganti 2021). Accordingly, we observed a significant increase in the mature form of the pro‐inflammatory cytokine IL‐1β in the HFD group, as shown by immunohistochemistry (Figure 7J,K) and ELISA (Figure 7L). Critically, BSE intervention effectively reversed all these changes, suppressing NLRP3 expression, reducing cleaved caspase‐1, and lowering mature IL‐1β levels (Figure 7D–L). Collectively, these results demonstrate that BSE mitigates HFD‐induced renal inflammation not only by reducing general pro‐inflammatory cytokines but also by inhibiting the activation of the NLRP3 inflammasome and its downstream signaling cascade.

## 4 Discussion

The present study demonstrates that dietary supplementation with BSE effectively ameliorates HFD‐induced renal injury in mice. This protection is characterized by attenuation of systemic dyslipidemia, renal lipid accumulation, oxidative stress, and inflammation. Our integrated analysis, combining pathway interrogation with untargeted lipidomics, reveals that BSE exerts these benefits through coordinated modulation of the AMPK/Nrf2 signaling axis and the NLRP3 inflammasome. Notably, we identified four specific lipid species (CAR 22:1, LPC 16:0, PI‐Cer 38:1; O3, NAE 26:5) that may serve as functional bridges between metabolic reprogramming and inflammation suppression. Together, these findings position BSE as a whole food‐derived dietary strategy against metabolic kidney disease and provide a multi‐target mechanistic framework that extends beyond the established Nrf2‐centric actions of sulforaphane.

The pathogenesis of HFD‐induced renal injury is increasingly understood as a self‐perpetuating cycle involving dysfunctional lipid metabolism, oxidative stress, and sterile inflammation (Mitrofanova et al. 2023). Our study confirms and connects these axes in our model: lipid overload suppresses AMPK activity, leading to impaired fatty acid oxidation and enhanced lipogenesis. This metabolic derangement promotes mitochondrial dysfunction and reactive oxygen species (ROS) overproduction, which in turn represses the Nrf2‐Keap1 antioxidant defense pathway (Tanase et al. 2022). The resulting redox imbalance, evidenced by depleted antioxidant enzymes and elevated lipid peroxidation, creates a tissue milieu conducive to the activation of the NLRP3 inflammasome, thereby amplifying inflammatory injury (Islamuddin and Qin 2024; Henedak et al. 2024).

The suppression of AMPK activity observed under HFD conditions is unlikely to be a passive event but rather the consequence of converging metabolic and inflammatory signals. Given that AMPKα2 is the predominant catalytic isoform in the kidney and is preferentially involved in fatty acid oxidation and mitochondrial biogenesis (Steinberg and Kemp 2009; Li et al. 2020), we selected this isoform as the molecular readout for AMPK activation in our metabolic model. Mechanistically, the suppression of AMPK activity under HFD conditions can be attributed to multiple interrelated factors. First, excessive lipid availability promotes mitochondrial β‐oxidation and ATP production, leading to an elevated ATP/AMP ratio, which directly reduces AMPK phosphorylation by the upstream kinase LKB1 (Koh et al. 2022). Second, accumulating fatty acid metabolites, such as acylcarnitines and ceramides, have been shown to activate protein phosphatase 2A (PP2A), which dephosphorylates AMPK at Thr172, thereby attenuating its activity (Ma et al. 2022). Interestingly, our lipidomics data revealed elevated CAR (22:1) levels in HFD‐fed kidneys, which may represent a lipid species contributing to this regulatory loop. Third, HFD‐induced endoplasmic reticulum stress and low‐grade inflammation can further inhibit AMPK phosphorylation (Zhou et al. 2025). Collectively, these mechanisms converge to establish a state of AMPK hypoactivity, which constitutes a critical early event in the transition from metabolic overload to renal injury.

Against this pathogenic backdrop, our findings reveal that BSE confers protection not through an isolated action but by appearing to orchestrate a coordinated intervention across this entire network. While SFN, a key isothiocyanate in BSE, is a well‐established Nrf2 agonist (Sun et al. 2017; Russo et al. 2018), our data demonstrate that BSE's mechanism may extend beyond Nrf2 activation. Crucially, BSE was simultaneously associated with activated AMPK signaling, reprogramming lipid metabolism at the transcriptional and functional levels, and suppressing the NLRP3 inflammasome cascade. The observed bioactivities of BSE can be rationally linked to its phytochemical composition. The UPLC‐HRMS analysis identified glucosinolates (including glucoraphanin and sinigrin) as major constituents, along with their hydrolysis products, isothiocyanates, and various phenolic compounds. Glucoraphanin is the natural precursor of SFN, a well‐characterized Nrf2 activator, while other isothiocyanates and phenolics may contribute additional antioxidant and anti‐inflammatory effects through complementary mechanisms (Vanduchova et al. 2019; Xu et al. 2018). Furthermore, certain carbohydrate conjugates and organic acids identified in BSE may influence energy metabolism and insulin sensitivity, potentially supporting AMPK activation (Bahadoran et al. 2012). Collectively, this complex phytochemical matrix supports a multi‐target mode of action, wherein the coordinated effects of multiple bioactive constituents, rather than a single compound, underlie the observed activation of AMPK/Nrf2 signaling and suppression of the NLRP3 inflammasome.

This multi‐target mode of action suggests a possible sequential and synergistic logic. By potentially activating AMPK, BSE may reset the metabolic foundation, alleviating the initial lipotoxic insult and reducing mitochondrial ROS production. AMPK activation is also known to phosphorylate and potentiate Nrf2 (Petsouki et al. 2022), which aligns with our observed concurrent upregulation of both pathways, leading to enhanced antioxidant capacity. The resultant mitigation of metabolic and oxidative stress collectively could dismantle the major triggers (e.g., ROS, ceramides) for NLRP3 inflammasome assembly (Moustakli et al. 2025; Swanson et al. 2019). Furthermore, SFN may directly inhibit NLRP3 activation (Kiser et al. 2021). Thus, BSE may intervene at multiple nodes, disrupting the vicious cycle at its origin (metabolism), its amplifier (oxidative stress), and its effector (inflammation), which could underlie its potent efficacy. Future mechanistic studies using pathway‐specific inhibitors or genetic approaches are warranted to directly test these causal relationships.

Our untargeted lipidomics data, when integrated with biochemical analyses, provide a molecular interface linking BSE's upstream modulation of AMPK/Nrf2 signaling to its downstream anti‐inflammatory outcome. The selective normalization of four key lipid species, including CAR (22:1), LPC (16:0), PI‐Cer (38:1; O3), and NAE (26:5), suggests that these lipids function not merely as biomarkers but as active mediators within the protective network. It is important to note that the number of significantly altered lipids was limited; however, these specific species were chosen based on their known roles as signaling molecules and their localization at critical nodes in metabolic‐inflammatory networks. These observations suggest that BSE does not broadly reverse all HFD‐induced lipid alterations but instead selectively normalizes a few hub lipid species that sit at critical intersections of metabolism and inflammation.

This targeted lipid remodeling mechanistically contributes to NLRP3 inflammasome suppression by directly counteracting its canonical activators. Specifically, the reduction in LPC (16:0), a damage‐associated molecular pattern (DAMP), likely attenuates the priming signal for NLRP3 expression (Liang et al. 2024). Concurrently, the decrease in CAR (22:1) indicates improved mitochondrial efficiency, which would curtail the release of mitochondrial ROS, a potent trigger for inflammasome assembly (Mu et al. 2022; Dambrova et al. 2022). Furthermore, the decline in PI‐Cer (38:1; O3) suggests a stabilization of sphingolipid metabolism, potentially reducing lysosomal membrane permeabilization and cathepsin release, another pathway implicated in NLRP3 activation (Dambrova et al. 2022). In parallel, the elevated NAE (26:5), an endogenous protective lipid, may engage anti‐inflammatory receptors to temper the inflammatory cascade (Mock et al. 2023; Murru et al. 2021).

Thus, these lipidomic shifts constitute a coordinated molecular intervention. They represent the functional downstream effectors of AMPK/Nrf2 activation and, in turn, actively reconfigure the renal microenvironment by quenching pro‐inflammatory triggers and enhancing protective signals (Baek et al. 2022; Zhang et al. 2019). This dual role, as both a consequence of cytoprotective signaling and a cause of inflammasome suppression, explains how BSE effectively disrupts the metabolic‐oxidative‐inflammatory axis in HFD‐induced nephropathy.

Therefore, our data support a model in which BSE, through its multi‐component composition, simultaneously engages complementary protective pathways. This multifaceted action, enhancing energy metabolism and antioxidant defenses while selectively normalizing pathogenic lipid species, collectively disrupts the propagative cycle of metabolic dysfunction, oxidative stress, and inflammation in HFD‐induced kidney injury. This coordinated strategy highlights the potential of whole‐food‐derived extracts like BSE in addressing complex multifactorial diseases and provides a solid mechanistic foundation for considering it as a valuable component of dietary strategies aimed at preventing metabolic‐stress‐induced kidney injury. The reduced plasma lipids (TC, TG, LDL‐C) and increased HDL‐C levels in BSE‐treated mice (Li, Wu, et al. 2025) are consistent with the observed attenuation of renal lipid accumulation.

While this study delineates a coherent mechanism for BSE's renoprotective effects, several limitations should be acknowledged. First, our findings are derived from a mouse model of HFD‐induced injury, and further validation in other models or species would strengthen the translational relevance of our conclusions. Second, urinary protein excretion and glomerular filtration rate (GFR) were not measured; future investigations incorporating these parameters will further strengthen the evaluation of renal function. Third, while we identified coordinated changes in AMPK/Nrf2 signaling and NLRP3 activation, these findings are primarily correlative; direct causal validation using pathway‐specific inhibitors or genetic knockdown models remains an important direction for future investigation. Fourth, this study used only male mice; given the potential sex differences in metabolic and inflammatory responses, future studies including both sexes are warranted. Fifth, the dose‐dependent effects of BSE, the potential contribution of other bioactive components beyond SFN, and the long‐term efficacy and safety of BSE supplementation requires further investigation in future preclinical and clinical studies. Addressing these points will be crucial for translating BSE from a promising dietary intervention into a practical strategy for renal health.

## Author Contributions

**Chunting Wu:** writing – original draft, formal analysis, investigation, writing – review and editing, data curation, validation. **Bangzhao Zeng:** investigation, formal analysis, writing – original draft, writing – review and editing, validation, data curation. **Xuexun Li:** investigation, formal analysis, writing – review and editing, writing – original draft, data curation, validation. **Xin Zhao:** methodology, software, writing – review and editing, data curation, validation. **Junyu Ma:** methodology, software, writing – review and editing, data curation, validation. **Mengxue Zhang:** writing – review and editing, software, visualization, data curation, validation. **Shuangshuang Zhao:** methodology, writing – review and editing, data curation, validation. **Chunmei Zhang:** visualization, supervision, writing – review and editing, data curation, validation. **Qin Gao:** methodology, funding acquisition, visualization, software, writing – review and editing, data curation, validation. **Fuli Ya:** supervision, funding acquisition, writing – review and editing, conceptualization, methodology, writing – original draft, data curation, validation.

## Funding

This work was supported by the National Natural Science Foundation of China (grant No. 82304122 and No. 82660752) and Yunnan Fundamental Research Projects (202401AT070081).

## Conflicts of Interest

The authors declare no conflicts of interest.

## Supporting information


**Table S1:** Primer sequences used for qRT‐PCR.
**Figure S1:** The percentage of all lipid subclasses was shown.
**Figure S2:** Base peak intensity (BPI) chromatograms of renal lipids. Representative BPI chromatograms in ESI− (A) and ESI+ (B) modes for the LFD, HFD, HFD + BSE groups and the pooled quality control (QC) sample.

## Data Availability

The data that support the findings of this study are available from the corresponding author upon reasonable request.
