# Supplementary material for: Broccoli Sprout Extract Restores Renal Lipid Metabolism in High‐Fat Diet‐Fed Mice Involving Modulation of AMPK/Nrf2 Signaling and the NLRP3 Inflammasome
Source: Food Sci Nutr. 2026 Sep 21;14(9):e72348. doi: 10.1002/fsn3.72348 (PMC13592130; doi:10.1002/fsn3.72348)
Supplement: Supplementary file 1 [file FSN3-14-e72348-s001.pdf]

Supporting information

Broccoli Sprout Extract Restores Renal Lipid Metabolism in High-Fat Diet-Fed Mice Involving Modulation of AMPK/Nrf2 Signaling and the NLRP3 Inflammasome

Chunting Wu[1#], Bangzhao Zeng[1#], Xuexun Li[1#], Xin Zhao[1], Junyu Ma[1], Mengxue Zhang[1], Shuangshuang Zhao[1], Chunmei Zhang[2], Qin Gao[3*], Fuli Ya[1*]

[1]*Department of Nutrition, School of Public Health, Dali University, Dali, Yunnan Province 671000, China;*

[2]*Department of Laboratory Teaching Center, School of Public Health, Dali University, Dali, Yunnan Province 671000, PR China;*

[3]*School of Public Health, Jining Medical University, Jining, Shandong Province 272000, PR China;*

[#]Chunting Wu, Bangzhao Zeng, and Xuexun Li contributed equally to this work.

*Corresponding authors:

Fuli Ya, MD, Ph.D

Department of Nutrition, School of Public Health, Dali University,

No. 22, Wanhua Road, Dali, Yunnan Province, PR China 671000;

Tel/Fax: 86-872-2257382;

E-mail: yafuli@yeah.net

Qin Gao, MD, Ph.D

School of Public Health, Jining Medical University,

No.133, Hehua Road, Jining, Shandong Province, PR China 272067;

E-mail: gqnutr@163.com

1. Supplementary methods

1.1 Animals and diets

All animal experiments were conducted in accordance with protocols approved by the Animal Care and Use Committee of Dali University (Permit Number: SYXK [Dian] 2018-0002). Six-week-old male C57BL/6J mice were procured from Kunming Chushang Technology Co., Ltd. (Kunming, Yunnan, China) and maintained under standard laboratory conditions, including a temperature-controlled environment (22–24 °C), a 12-hour light/dark cycle, and *ad libitum* access to food and water. Following a two-week acclimatization period, the mice were randomly allocated into four dietary groups (n=10 per group) for a 12-week intervention: a low-fat diet group (LFD, 10% fat), an LFD group supplemented with broccoli sprout extract (LFD+BSE, 600 mg/kg diet), a high-fat diet group (HFD, 45% fat), and an HFD group supplemented with BSE (HFD+BSE, 600 mg/kg diet) (Li et al., 2025). All diets were obtained from Medicience Ltd. (Jiangsu, China), and their detailed compositions have been described previously (Li et al., 2025). The BSE, administered as a freeze-dried powder derived from fresh broccoli sprouts (*Brassica oleracea* L. var. *botrytis* L.), was purchased from Haosen Biotechnology Co., Ltd (Xi'an, Shanxi, China) and homogenously blended into the respective diets at a concentration of 0.06% (w/w), a dosage consistent with previously established protocols (Lei et al., 2018). At the start of the intervention, no significant differences in body weight were observed among the four groups (LFD: 17.63 ± 0.75 g; LFD+BSE: 17.49 ± 0.84 g; HFD: 17.58 ± 0.79 g; HFD+BSE: 17.60 ± 0.99 g; $P > 0.05$), confirming successful randomization. Body

weight and food intake were monitored and recorded throughout the study on a weekly basis.

1.2 BSE preparation

BSE was prepared from fresh broccoli sprouts (*Brassica oleracea* L. var. *botrytis* L.). Briefly, broccoli seeds were germinated and cultivated under controlled conditions. The harvested fresh sprouts were freeze-dried and ground into a fine powder. The powder was subjected to solvent extraction with 70% aqueous ethanol (1:20, w/v) at 60 °C for 2 h under continuous agitation. The extract was filtered through a 0.45 μm membrane, and the filtrate was concentrated under reduced pressure at 50 °C using a rotary evaporator to obtain a crude paste. The paste was then redissolved in distilled water and spray-dried at an inlet temperature of 160 °C and an outlet temperature of 80 °C. The resulting powder was sieved (80 mesh), homogenized, vacuum-packed in light-protective bags, and stored at –20 °C until use.

1.3 Analysis of phytochemical composition in BSE by UPLC-HRMS

1.3.1 Hydrophilic Compounds

The phytochemical composition in BSE was analyzed using ultra-performance liquid chromatography–high resolution mass spectrometry (UPLC-HRMS). For hydrophilic compounds, 50 mg of BSE powder was extracted with 400 μL ice-cold methanol, kept at -20°C for 30 min, and centrifuged at 20,000 × *g* for 15 min. The supernatant was transferred and centrifuged again under the same conditions before UPLC-HRMS analysis. Separation was performed on an ACQUITY BEH Amide column (100 mm × 2.1 mm, 1.7 μm) with mobile phase A (5 mM ammonium acetate + 0.1% ammonium

hydroxide in water) and B (acetonitrile). The gradient was: 95% B (0–0.5 min), 95%→75% B (0.5–4.0 min), 75%→40% B (4.0–7.0 min), 40% B (7.0–9.0 min), 40%→95% B (9.0–9.1 min), and 95% B (9.1–12.0 min) at 0.5 mL/min. MS detection used an Orbitrap Exploris 120 with spray voltages of ±3800 V, resolution of 60,000, and m/z range 70–1050.

1.3.2 Lipophilic Compounds

For lipophilic compounds, 50 mg of BSE was extracted with 400 μL MTBE and 80 μL methanol, vortexed, and centrifuged at 3000 rpm for 15 min. The supernatant was dried, reconstituted in 200 μL dichloromethane:methanol (1:1), and centrifuged again before analysis. An ACQUITY UPLC CSH C18 column (100 mm × 2.1 mm, 1.7 μm) was used with mobile phase A (acetonitrile:water = 60:40 with 10 mM ammonium formate and 0.1% formic acid) and B (isopropanol:acetonitrile = 90:10 with 10 mM ammonium formate and 0.1% formic acid). The gradient was: 30% B (0–0.4 min), 30%→45% B (0.4–1.0 min), 45%→60% B (1.0–3.5 min), 60%→75% B (3.5–5.0 min), 75%→90% B (5.0–7.0 min), 90%→100% B (7.0–8.5 min), 100% B (8.5–8.6 min), 100%→30% B (8.6–8.61 min), and 30% B (8.61–10.0 min) at 0.3 mL/min. MS analysis used a Q-Exactive Plus with spray voltages of ±3800 V, resolution of 70,000, and DDA mode (m/z 100–1500). Quality control samples were prepared from all extracts and analyzed periodically to ensure system stability.

1.4 Blood and kidney tissue collection

At the end of the 12-week dietary intervention, mice were fasted for eight hours and subsequently anesthetized via an intraperitoneal injection of 10% chloral hydrate

(0.02 mL/g body weight). Blood was collected from the heart using a syringe prefilled with 3.8% acid citrate dextrose (ACD; at a 1:10 ACD-to-blood volume ratio) as an anticoagulant. To obtain plasma, the anticoagulated blood was subjected to a series of centrifugations: first at 300 × *g* for 2 min at 37°C to prepare platelet-rich plasma (PRP), then the PRP was centrifuged at 500 × *g* for 3 min to pellet platelets. The resulting supernatant was further centrifuged at 12,000 × *g* for 10 min at 4°C to obtain platelet-poor plasma, which was aliquoted and stored at -80°C for subsequent biochemical analyses. Following blood collection, the thoracic cavity was opened, and systemic perfusion was performed with saline via the left ventricle to clear residual blood from the circulation. Kidneys were then excised, weighed, and processed for further studies.

1.5 Determination of serum biochemical parameters and renal lipid contents

Serum creatinine (SCr), uric acid (UA), and blood urea nitrogen (BUN) levels were measured using an automatic biochemical analyzer (Beckman Instruments, Inc., USA). Renal lipid levels, including total cholesterol (TC), triglycerides (TG), and free fatty acids (FFA), were determined with commercially available assay kits according to the manufacturers' instructions. Kits for TC and TG were obtained from Nanjing Jiancheng Bio Co. (Nanjing, China), and the FFA assay kit was sourced from Sangon Biotech Co., Ltd. (Shanghai, China).

1.6 Measurement of renal oxidative stress and inflammatory markers

Oxidative stress and inflammatory markers in kidney tissues were evaluated using commercially available assay kits. Approximately 100 mg of renal tissue was

homogenized on ice and centrifuged at 10,000 × *g* for 10 min at 4°C. The supernatant was collected for protein quantification and subsequent analyses. Oxidative stress parameters, including total antioxidant capacity (T-AOC), superoxide dismutase (SOD) activity, and malondialdehyde (MDA) content, were measured strictly according to the manufacturers' instructions (T-AOC and SOD kits from Beijing Boxbio Science & Technology Co., Ltd., Beijing, China; MDA kit from Nanjing Jiancheng Bioengineering Institute, Nanjing, China). Absorbance was recorded at 593 nm (T-AOC), 560 nm (SOD), and 532 nm (MDA). Results were expressed as μmol/mg protein for T-AOC, U/mg protein for SOD activity, and nmol/g tissue for MDA. Levels of the inflammatory cytokines TNF-α and IL-1β were quantified using specific ELISA kits (TNF-α from Wuhan Yunclone Technology Co., Ltd., Wuhan, China; IL-1β from Beijing Solarbio Science & Technology Co., Ltd., Beijing, China) according to the provided protocols, with absorbance measured at 450 nm. Concentrations were expressed as pg per mg of total protein.

1.7 Western blot analysis

Kidney tissues were homogenized in RIPA lysis buffer containing protease and phosphatase inhibitors (Beyotime Biotechnology, Shanghai, China) using a tissue homogenizer (Servicebio, Wuhan, China). The homogenates were centrifuged at 12,000 × *g* for 15 min at 4°C, and the resulting supernatants were collected for protein concentration determination using a BCA protein assay kit (KeyGEN Biotechnology, Nanjing, China). Proteins (30 μg per sample) were separated by SDS-PAGE and transferred onto PVDF membranes (Millipore, USA) (Li et al., 2025; Ma et al., 2026;

Zhou et al., 2023). After blocking with 5% BSA for 1.5 h, the membranes were incubated overnight at 4°C with primary antibodies against the following proteins: Fibronectin (1:1000), TGF-β1 (1:1000), SREBP1 (1:1000), SCD (1:1000), SIRT1 (1:1000), PPARα (1:500), CPT1A (1:1000), PGC1α (1:1000), phospho-AMPKα2 (Thr172) (1:2000), Nrf2 (1:2000), TNF-α (1:500), IL-6 (1:1000), NLRP3 (1:1000), cleaved Caspase-1 (1:2000). GAPDH (1:10000) and β-actin (1:10000) were used as loading controls. Following incubation with appropriate HRP-conjugated secondary antibodies, protein bands were visualized using an ECL detection system. Band intensities were quantified with ImageJ software (version 1.37v; NIH, USA) and normalized to the corresponding loading control. Loading controls (GAPDH or β-actin) were selected based on molecular weight compatibility with each target protein to avoid signal overlap. Both housekeeping proteins were confirmed to be stably expressed across all experimental groups.

1.8 Untargeted lipidomics analysis by UPLC-TOF/MS

1.8.1 Sample preparation

Kidney tissue samples (50 mg wet weight) were homogenized in 200 μL of ice-cold 50% methanol using a Precellys 24 homogenizer equipped with stainless steel grinding beads (two cycles of 30 s at 6000 rpm, 4°C). Lipids were extracted by adding 600 μL of ice-cold methyl tert-butyl ether (MTBE) to the homogenate, followed by vortex mixing for 30 min at 4°C. After centrifugation (3000 × *g*, 15 min, 4°C), 200 μL of the upper organic phase was collected and lyophilized. The dried extract was reconstituted in 200 μL of dichloromethane:methanol (1:1, v/v) containing 0.01%

butylated hydroxytoluene (BHT). Following a final centrifugation (3000 × *g*, 15 min, 4°C), the supernatant was transferred to an injection vial for UPLC-HRMS analysis. A quality control (QC) sample was prepared by combining equal aliquots from all individual samples.

1.8.2 Lipidomics conditions

Chromatographic separation was achieved using an ACQUITY UPLC CSH C18 column (100 mm × 2.1 mm, 1.7 μm) maintained at 40°C. The mobile phase consisted of (A) 10 mM ammonium formate with 0.1% formic acid in acetonitrile:water (60:40, v/v) and (B) 10 mM ammonium formate with 0.1% formic acid in isopropanol:acetonitrile (90:10, v/v). A gradient elution was performed at a flow rate of 0.3 mL/min as follows: 30% B (0–0.4 min), 30–45% B (0.4–1.0 min), 45–60% B (1.0–3.5 min), 60–75% B (3.5–5.0 min), 75–90% B (5.0–7.0 min), 90–100% B (7.0–8.5 min), hold at 100% B (8.5–8.6 min), and re-equilibration at 30% B (8.61–10.0 min). The injection volume was 4 μL. Mass spectrometry was performed on a TripleTOF 6600 system (SCIEX) equipped with a DuoSpray ion source, operating in both positive and negative electrospray ionization modes. The key parameters were set as: ion spray voltage, ±5000 V; source temperature, 500°C; curtain gas, 30 psi; nebulizer and heater gases, 60 psi. Data were acquired in information-dependent acquisition mode, consisting of a full MS scan (*m/z* 50–2000) followed by MS/MS scans of the top 12 most intense ions.

1.8.3 Quality control, data processing and analysis

System stability was monitored by injecting QC samples at regular intervals (every six experimental samples). Quality assurance was based on the following criteria: a

coefficient of variation (CV) of less than 30% for features in QC samples, an inter-QC correlation coefficient (r) greater than 0.95, and a dispersion of less than 2% total variance in principal component analysis. Raw data were converted to mzML format and processed using XCMS for peak detection, alignment, and annotation. Metabolite identification was performed by matching accurate mass and retention time against the KEGG and HMDB databases. Statistical analysis included one-way ANOVA with Tukey's post hoc test, with *P*-values adjusted for false discovery rate. Supervised partial least squares-discriminant analysis was employed to identify discriminatory lipid species, with a variable importance in projection score greater than 1.0 considered significant.

Supplementary references

Lei P., Zhao W., Pang B., et al. 2018. Broccoli Sprout Extract Alleviates Alcohol-Induced Oxidative Stress and Endoplasmic Reticulum Stress in C57BL/6 Mice. *Journal of agricultural and food chemistry,* 66, 5574-5580.

Li C., Wu Y., Liu K.Y., et al. 2024. Cornus officinalis vinegar alters the gut microbiota, regulating lipid droplet changes in nonalcoholic fatty liver disease model mice. *Food & Medicine Homology,* 1, 9420002.

Guo L.M., Zhang W.Y., Wang L.W., et al. 2025. Anti-fibrotic effects of marein-enriched Coreopsis tinctoria flavonoids on CCl4-induced hepatic fibrosis in rats via blocking the TGF-β1/Smad signaling pathway. *Food Science and Human Wellness,* 14, 9250499.

Li M.Y., Zhang C.M., Ma J.Y., et al. 2025. Curcumin Attenuates Liver Injury by Modulating the AGE–RAGE Axis and Metabolic Homeostasis in High-Fat Diet/Streptozotocin-Induced Type 2 Diabetic Mice. *Front Nutr,* 12, 1710380.

Zhang S.L., Gong L.H, Yao Y.X., et al. 2025. Mechanisms of Phillygenin and Forsythiaside A in Liver Fibrosis Based on Hepatocyte-Derived Exosomes. *Food Science and Human Wellness*. https://doi.org/10.26599/FSHW.2025.9250840

Li W.Q., Wu C.T., Zhou X.Y., et al. 2025. Sulforaphane Attenuates CD36-mediated Platelet Hyperreactivity through Modulating cAMP/PKA/NOX2 Signaling in Hyperlipidemic Conditions. *Food Science and Human Wellness,* 14, 9250165.

Chen X.M., Zeng X., Deng C.F., et al. 2026. Hepatoprotective effects of sweet tea and trilobatin against alcohol-induced acute liver injury in mice. *Food & Medicine Homology*, 3, 9420109.

Ma Y.J., Hu J.Q., Bi X.Y., et al. 2026. Panax Notoginseng Saponins Attenuate Oxidative Stress-mediated Platelet Apoptosis in Hyperlipidemic Conditions via Up-regulating cAMP/PKA Signaling Pathway in vitro and in vivo. *Journal of Future Foods,* 6, 803-817.

Zhou X.Y., Huang X.H., Wu C.T., et al. 2023. Sulforaphane attenuates glycoprotein VI-mediated platelet mitochondrial dysfunction through up-regulating the cAMP/PKA signaling pathway in vitro and in vivo. *Food & function,* 14, 3613-3629.

2. Supplementary tables, figures and legends

Supplementary Table S1. Primer sequences used for qRT-PCR.

Genes	Primer	Sequences
AMPKα2	Forward	5’-CTGAAGCCAGAGAATGTGCTGC-3’
	Reverse	5’-GAGATGACCTCAGGTGCTGCAT-3’
HO-1	Forward	5’-CACTCTGGAGATGACACCTGAG-3’
	Reverse	5’-GTGTTCCTCTGTCAGCATCACC-3’
NQO1	Forward	5’-GCCGAACACAAGAAGCTGGAAG-3’
	Reverse	5’-GGCAAATCCTGCTACGAGCACT-3’
NLRP3	Forward	5’-TCACAACTCGCCCAAGGAGGAA-3’
	Reverse	5’-AAGAGACCACGGCAGAAGCTAG-3’
Nrf2	Forward	5’-CAGCATAGAGCAGGACATGGAG-3’
	Reverse	5’-GAACAGCGGTAGTATCAGCCAG-3’
GAPDH	Forward	5’-CATCACTGCCACCCAGAAGACTG-3’
	Reverse	5’-ATGCCAGTGAGCTTCCCGTTCAG-3’

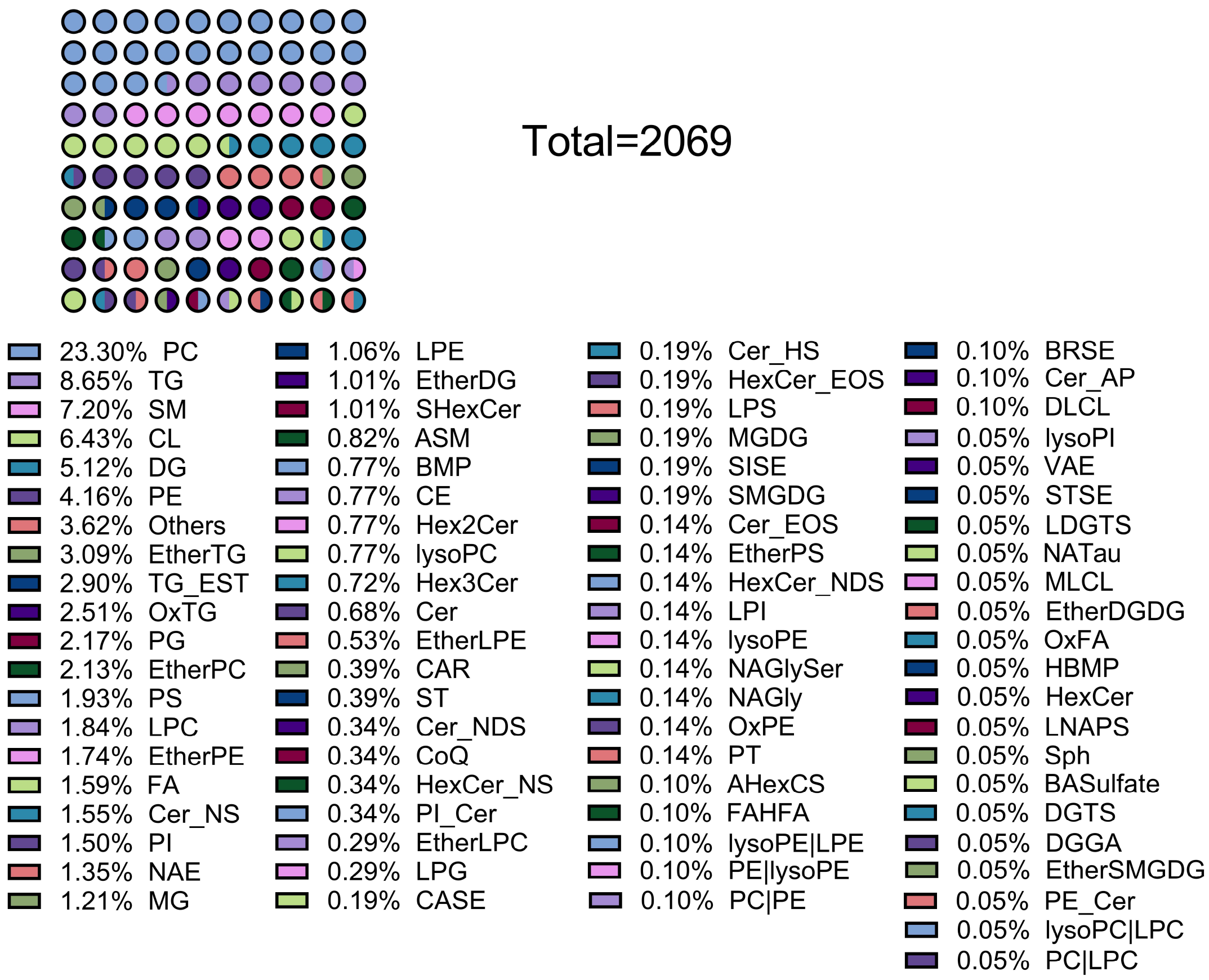

Supplementary Figure S1. The percentage of all lipid subclasses was shown.

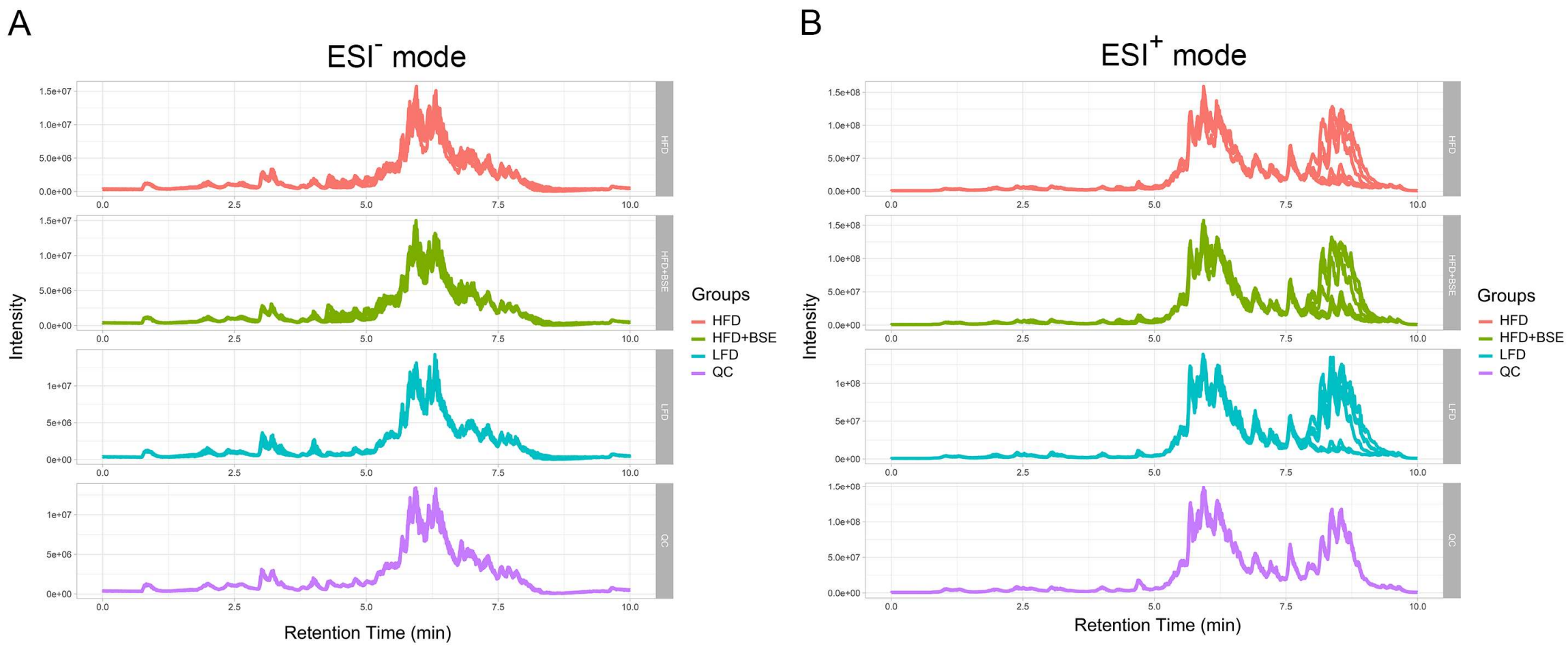

Supplementary Figure S2. Base peak intensity (BPI) chromatograms of renal lipids. Representative BPI chromatograms in ESI$^-$ **(A)** and ESI$^+$ **(B)** modes for the LFD, HFD, HFD+BSE groups and the pooled quality control (QC) sample.
